# Claudin-1 overexpression in intestinal epithelial cells enhances susceptibility to adenamatous polyposis coli-mediated colon tumorigenesis

**DOI:** 10.1186/1476-4598-13-167

**Published:** 2014-07-06

**Authors:** Jillian L Pope, Rizwan Ahmad, Ajaz A Bhat, Mary K Washington, Amar B Singh, Punita Dhawan

**Affiliations:** 1 Department of Veterans Affairs, Vanderbilt University Medical Center, Nashville, TN 37232, USA; 2 Departments of Cancer Biology, Vanderbilt University Medical Center, Nashville, TN 37232, USA; 3 Departments of Surgery and Cancer Biology, Vanderbilt University Medical Center, Nashville, TN 37232, USA; 4 Pathology, Vanderbilt University Medical Center, Nashville, TN 37232, USA; 5 Medicine, Vanderbilt University Medical Center, Nashville, TN 37232, USA

**Keywords:** Claudin-1, Colorectal cancer, IL23, Bacteria, Muc2

## Abstract

**Background:**

The tight junction protein Claudin-1, a claudin family member, has been implicated in several gastro-intestinal pathologies including inflammatory bowel disease (IBD) and colorectal cancer (CRC). In this regard, we have demonstrated that claudin-1 expression in colon cancer cells potentiates their tumorigenic ability while *in vivo* expression of claudin-1 in the intestinal epithelial cells (IECs) promotes Notch-activation, inhibits goblet cell differentiation and renders susceptibility to mucosal inflammation. Notably, a key role of inflammation in colon cancer progression is being appreciated. Therefore, we examined whether inflammation plays an important role in claudin-1-dependent upregulation of colon carcinogenesis.

**Methods:**

APC^min^ mice were crossed with Villin-claudin-1 transgenic mice to generate APC-Cldn1 mice. H&E stained colon tissues were assessed for tumor number, size and histological grade. Additionally, microarray and qPCR analyses of colonic tumors were performed to assess molecular changes due to claudin-1 expression. APC-Cldn1 and APC^min^ controls were assessed for colonic permeability via rectal administration of FITC-dextran, and bacterial translocation via qPCR analysis of 16S rDNA.

**Results:**

Claudin-1 overexpression in APC^min^ mice significantly increased (~4-fold) colonic tumor growth and size, and decreased survival. Furthermore, transcriptome analysis supported upregulated proliferation, and increased Wnt and Notch-signaling in APC-Cldn1 mice. APC-Cldn1 mice also demonstrated inhibition of mucosal defense genes while expression of pro-inflammatory genes was sharply upregulated, especially the IL-23/IL-17 signaling. We predict that increased Notch/Wnt-signaling underlie the early onset of adenoma formation in APC-Cldn1 mice. An increase in mucosal permeability due to the adenomas and the inherent barrier defect in these mice further facilitate bacterial translocation into the mucosa to induce inflammation, which in turn promote the tumorigenesis.

**Conclusion:**

Taken together, these results confirm the role of claudin-1 as a promoter of colon tumorigenesis and further identify the role of the dysregulated antigen-tumor interaction and inflammation in claudin-1-dependent upregulation of colon tumorigenesis.

## Background

Claudin-1 is a member of the claudin family of tight junction proteins whose traditional roles involve maintenance of the epithelial barrier function. However, in recent years many claudins have been shown to be important players in several types of cancers, in capacities beyond barrier regulation, when their expression levels/patterns are altered. In most of these studies however, loss of the TJ proteins have contributed to the deregulation of the mechanical aspects of tumor progression such as migration [1], and invasion [2,3]. By contrast, we and others have shown that the claudin-1 expression is upregulated in human colon cancer [4] and that modulation of claudin-1 expression positively regulates the tumor growth and metastasis in xenograft models using colon cancer cells. However, it remains unclear if modulation of claudin-1 expression even in normal colonic epithelial cells would serve a tumor promoting role, under conditions permissive of colon cancer growth, and the underlying molecular mechanisms.

Importantly, in recent studies, we have demonstrated a role for claudin-1 in the maintenance of normal intestinal homeostasis whereby intestinal epithelial cell (IEC)-specific constitutive expression of claudin-1 (Villin-claudin-1 Tg mice; Cld-1Tg) altered goblet cell differentiation by promoting Notch activation [5]. Importantly, Cl-1Tg mice also demonstrated enhanced severity of Dextran sodium sulfate (DSS)-induced colitis and impaired recovery from colitis-induced epithelial injury, which was attributed to the decreased mucosal protection due to the loss of the primary component of the goblet cells and anti-microbial defense, Mucin-2. These findings supported a previously reported connection between inflammation and claudin-1 expression using specimens from patients with active IBD and colitis-associated cancer [6,7].

To determine whether IEC-specific claudin-1 overexpression would also promote colon tumorigenesis, we generated APC-Cldn1 mice by crossing Cld-1Tg mice with APC^Min^ mice, the commonly used mouse model of intestinal tumorigenesis. The APC^Min^ mouse model replicates the common mutation inherited in Familial Adenamatous polyposis disease (FAP), which predisposes patients to spontaneous colorectal cancer. Notably, the APC^Min^ mice regularly develop adenomas of the small intestine however they rarely develop those of large bowel origin [8]. Consequently, these mice are widely used to study the role of the specific genes of interest in the regulation of colorectal cancer in conjunction with the APC mutation.

Here, we show that claudin-1 overexpression in APC^Min^ mice significantly increases colon tumor growth as well as frequency while decreasing the mice survival. In concurrence with our previous reports, tumors in APC-Cldn1 mice demonstrate elevated levels of Wnt- and Notch-signaling. Furthermore, an upregulated pro-inflammatory gene signature including the IL-23/IL-17-signaling and suppressed anti-microbial defense mechanisms marked these tumors. Notably, these dysregulations associated with an increase in mucosal permeability and bacterial translocation in APC-Cldn1 mice, which is reported to upregulate IL-23/IL-17 signaling [9], and may aid in the promotion of colon cancer. Taken together, our current studies provide a clear insight into the role of claudin-1 protein in the regulation of colorectal cancer potentially by upregulating the Notch- and Wnt-signaling and mucosal inflammation.

## Results

### Claudin-1 overexpression increases colon tumorigenesis and decreases mice survival

**Figure 1:**
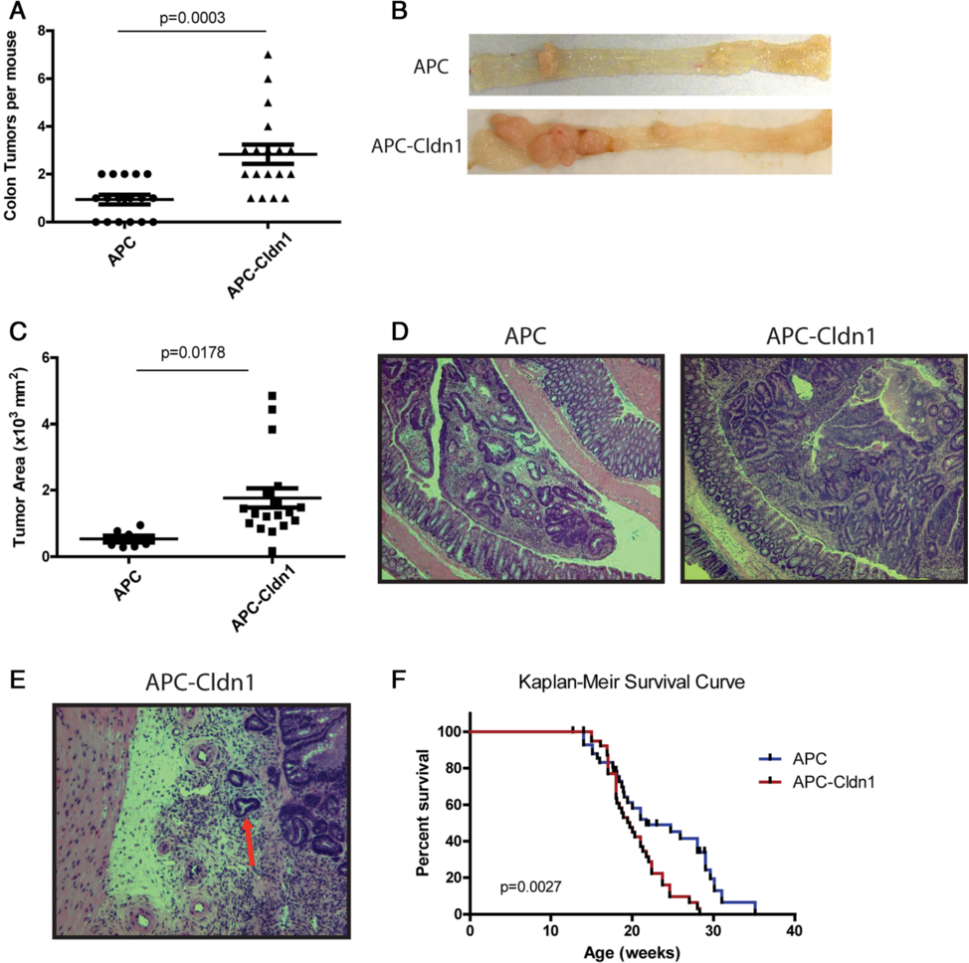
**IEC-specific constitutive expression of claudin-1 in APC**^**Min **^**mice increases colon tumorigenesis and decreases survival. (A)** APC^Min^ mice were crossed with Cld-1Tg mice and colon tumors were quantitated from littermate of APC^Min^ and APC-Cldn1 mice (n = 18 APC mice, n = 17 APC-Cldn1 mice; p = 0.0003). **(B)** Representative images of the colon from APC^Min^ and APC-Cldn1 mice showing increased sporadic colon tumors. **(C)** Tumor area was measured in APC^Min^ and APC-Cldn1 mice (p = 0.0178). **(D)** Representative H&E staining. **(E)** Invading carcinoma in APC*-*Cldn1 mouse. **(F)** Kaplan Meir survival curve demonstrating survival of APC^Min^ (n = 43) and APC-Cldn1 (n = 40) mice (p = 0.0027). Values are mean ± S.E.M.

**Figure 2:**
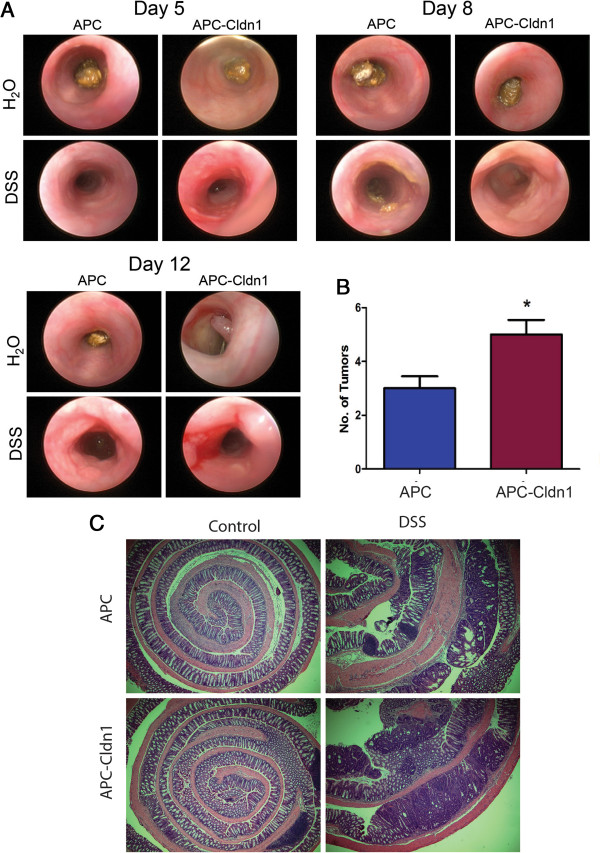
**Claudin-1 overexpression promotes inflammation driven colon tumorigenesis. (A)** Endoscopic images were obtained from APC^Min^ and APC-Cldn1 mice under conditions of regular drinking water (control) (n = 3) and 2% DSS w/v in drinking water (treated) (n = 5) mice at the indicated time points, following 7 days of DSS administration. **(B)** Tumor number/mice in DSS-treated APC^Min^ *versus* APC-Cldn1 mice (p = 0.022) **(C)** Representative H&E images. Values are mean ± S.E.M. *p < 0.05.

Increased claudin-1 expression has been frequently observed in colon cancer, however the consequences of the *in-vivo* upregulation in colonic epithelial cells have not been investigated. To determine the role of claudin-1 in colon tumorigenesis, we crossed Villin-Claudin-1-Tg mice with APC^Min^ mice (APC) to generate APC^Min^-Villin-Claudin-1 (APC-Cldn1) mice. We observed robust expression of claudin-1, localized to the membrane, in the colon of APC-Cldn1 mice compared to APC mice (Additional file 1: Figure S1). APC mice characteristically develop adenomas in the small intestine with little to no tumor occurrence in the colon [8]. In our studies, APC-Cldn1 (n = 18) mice developed colonic tumors at a significantly higher frequency (p = 0.0003) than APC mice (n = 18) (Figure 1A). Endoscopy of mouse colon at 10 weeks of age showed that APC-Cldn1 mice developed colonic tumor at this early age compared to APC mice (Figure 2A, Day 12, water treated group). Further, the tumors in APC-Cldn1 mice colon appeared larger than the APC mice colon tumors (p = 0.0178; measured using imaging analysis software (Figure 1C,D). The histological analysis further demonstrated that the tumors in APC-Cldn1 mice colon were less differentiated and high grade compared to the APC^Min^ mice (p = 0.0007) (Figure 1D, Table 1). Notably, it is rare that adenomas of APC^Min^ mice, originating from the colon or small bowel progress to invasive adenocarcinoma, yet we were able to detect an incident of invasion in the APC-Cldn-1 mice (Figure 1E, Table 1). Through routine care and observation of the mice, we also noticed that APC-Cldn1 mice began showing signs of morbidity much sooner than APC^Min^ mice. The average life span of an APC^Min^ mouse is approximately six months. To determine if there was a significant difference in survival, we plotted a Kaplan Meir curve and found that APC-cldn1 mice (n = 40) have a statistically significant reduced survival time to four months (p = 0.0027) compared to APC^Min^ mice (n = 43) (Figure 1F). As mentioned previously, multiple adenoma formation is restricted to the small intestine in the APC model and is thought to attribute to their limited life span. Therefore we thought it important to assess whether tumors of the small intestine also progress with claudin-1 overexpression. We found no significant difference in the number of intestinal tumors between the APC^Min^ and APC-Cldn1 mice. However, an increased trend was observed and the intestinal tumors in APC-Cldn1 mice were in general, advanced and displayed high-grade dysplasia (Additional file 2: Figure S2 and Additional file 3: Table S1). Taken together, these results suggested that increased claudin-1 expression enhances susceptibility to tumor development in the colon of APC^Min^ mice as well as contributes to the tumor progression.

**Table 1:** Comparative histological analysis of colon tumors

**Genotype**	**No. mice with tumors**	Average no. tumors per mouse*	Total no. mice w/ HGD**	**Total no. invasive adenocarcinomas**	**Total no. of adenomas**
APC (*n = 18*)	15	1.28	2	0	22
APC-Cldn1 (*n = 16*)	16	2.76	11	1	45

*Abbreviation*: *HGD* high grade dysplasia.

*p = 0.003.

**Logistic regression for likelihood of developing HGD p = 0.0007.

Colon tumors were quantitated from APC^Min^ and APC-Cldn1 mice and were classified as high grade dysplasia (HGD), adenomas and invasive adenocarcinomas.

### APC-Claudin-1 tumors have increased Wnt/Notch signaling

**Figure 3:**
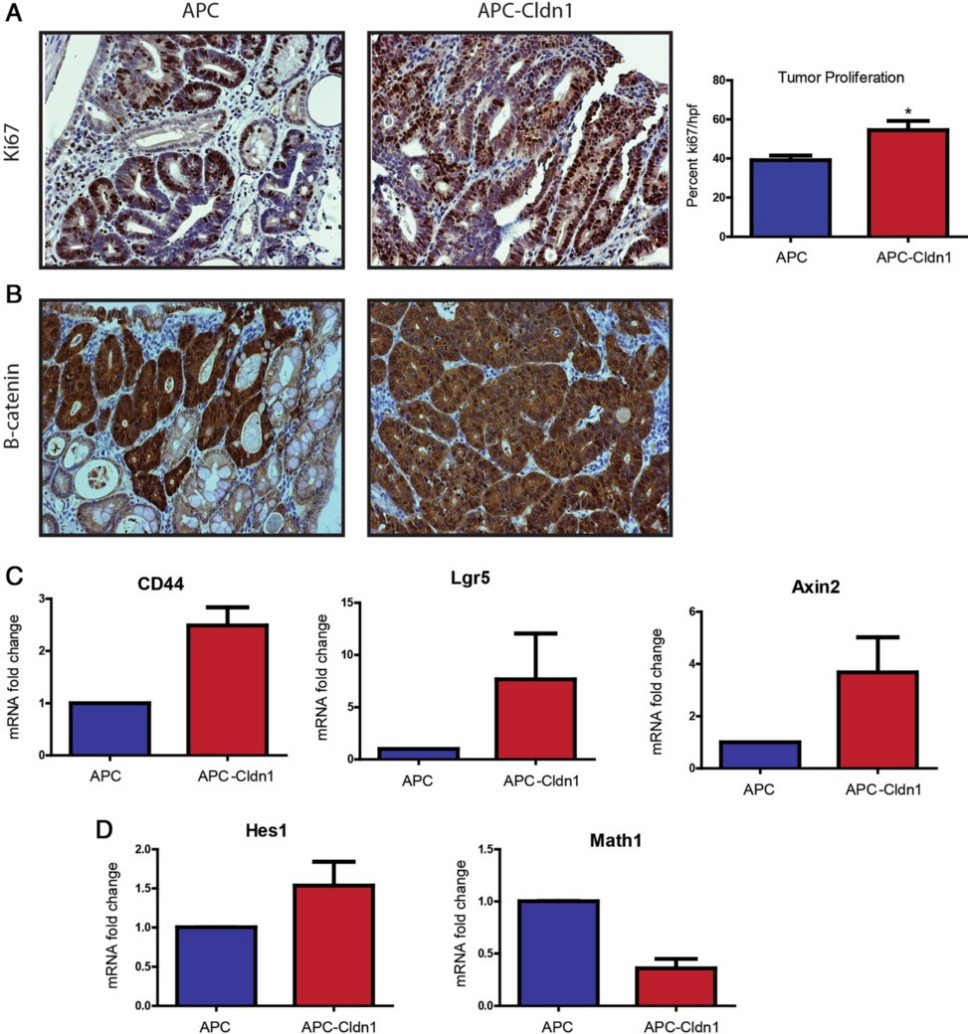
**Increased proliferation and upregulated Wnt- and Notch-Signaling contributes to the increased tumorigenesis in APC-Cldn1 mice. (A)** Tumors from APC^Min^ and APC-Cldn1 mice were immunostained using anti-Ki67 antibody to quantitate the proliferation, (n = 9 APC, n = 10 APC-Cld-1mice) p = 0.0125. **(B)** Activation of Wnt signaling was determined by immunostaining for β-catenin using anti-β-catenin antibody. The nuclear/cytoplasmic β-catenin staining is increased in APC-Cldn1 mice tumors compared to APC^Min^ mice. **(C)** qRT-PCR analysis of Wnt target genes and **(D)** Notch target genes in tumors of APC^Min^ and APC-Cldn1 mice (n = 3 mice each group). Values are mean ± S.E.M. *p < 0.05.

An increase in tumor size or number usually results from an increase in proliferation and/or an associated decrease in apoptosis. Therefore to assess proliferation in these tumors, we performed immunostaining for Ki67, a well-known marker of cellular proliferation. We quantified a significant increase (p = 0.0125) in the proliferation in APC-Cldn1 mice tumors compared to the APC^Min^ mice (Figure 3A). Immunostaining using anti-cleaved caspase-3 antibody however suggested no significant differences in the apoptosis (Additional file 4: Figure S3). As claudin-1 is a downstream target of Wnt signaling [10], and tumors that arise from the loss of APC have constitutive Wnt activation, we decided to examine the colonic tumors in each of these mice for potential upregulation of the Wnt/β-catenin pathway. We immunostained tumors for β-catenin, the primary effector of Wnt-activation, to assess for nuclear/cytoplasmic staining, which is an indicator of activated β-catenin. APC-Cldn1 tumors showed a noticeable increase in nuclear and cytoplasmic staining compared to that of APC^Min^ tumors (Figure 3B). To further assess Wnt upregulation, we performed qRT-PCR analysis using total RNA isolated from the colonic tumors of APC^Min^ and APC-Cldn1 and detected increased mRNA expression of the established Wnt-target genes CD44 (p = 0.0159), Lgr5 (5-fold increase), and Axin2 (3-fold increase) (Figure 3C). Previously, we have shown that IEC-specific claudin-1 expression in mice induces Notch-signaling [5]. Additionally, several studies have shown a role for the Notch pathway in colon tumorigenesis and targeted therapy [11,12]. Indeed, we did find increased Hes1 and decreased Math1 expression in the tumors in APC-Cldn1 mice colon (p < 0.0001), suggesting upregulation of the Notch-signaling (Figure 3D). Taken together, we concluded that claudin-1 expression aids in colon tumor progression via upregulation of the Notch- and Wnt-signaling pathways.

### APC-Claudin-1 tumors demonstrate increased inflammation

To further assess global changes that occur as a result of claudin-1 upregulation, we performed transcriptome analysis of colonic tumors from APC^Min^ and APC-Cldn1 mice to identify differentially expressed genes (DEGs). Table 2 shows the list of the selected genes that were upregulated or downregulated by a factor of at least 1.5 fold or greater in APC-Cld1 mice (*versus* APC^Min^ mice). In accordance with the data shown above, tumors of APC-Cldn1 mice showed altered expression of genes correlative with increased Wnt and Notch activities.

**Table 2:** **Microarray analysis of APC-Cldn1 *versus *** **APC**^**Min **^**mice tumors**

***Signaling gene symbol***	***p-value***	***Fold change***
***A. Wnt signaling related genes***		
*Tcf4*	*0.068*	*1.581*
*Lef1*	*0.017*	*2.238*
*Fzd10*	*0.016*	*2.219*
*Axin2*	*0.040*	*1.693*
*Wnt6*	*0.022*	*1.886*
*Wnt10a*	*0.125*	*1.911*
*Sox17*	*0.064*	*2.694*
*Ephb6*	*0.029*	*2.527*
***B. Notch signaling related genes***		
*Tcf4*	*0.068*	*1.581*
*Lef1*	*0.017*	*2.238*
*Fzd10*	*0.016*	*2.220*
*Axin2*	*0.040*	*1.694*
*Mmp9*	*0.042*	*1.628*
*Atoh1*	*0.047*	*-3.487*
*Klf4*	*0.010*	*-2.495*
*Muc2*	*0.112*	*-2.141*
*Muc3*	*0.002*	*-6.700*
*Muc4*	*0.345*	*-1.590*
*Tff3*	*0.184*	*-1.789*
***C. lnflammation/lmmune response genes***		
*lL23a, p19*	*0.011*	*2.250*
*CxcI5*	*0.060*	*2.392*
*CxcI9*	*0.134*	*2.394*
*CxcI2*	*0.462*	*1.548*
*Mmp9*	*0.042*	*1.628*
*Nfat5*	*0.031*	*1.515*
*Pla2g5*	*0.002*	*2.796*
*Retn1b*	*0.045*	*10.865*
*Lcn2*	*0.001*	*1.894*
*Ccl6*	*0.075*	*1.658*
*Ccl28*	*0.002*	*5.352*
*Ccl3*	*0.001*	*1.894*
*IL1b*	*0.048*	*1.825*
*Csf3r*	*0.056*	*1.567*
*Akt2*	*0.001*	*1.541*
*H2-AA*	*0.017*	*1.764*
*H2-Eb1*	*0.051*	*1.666*
*CD244*	*0.010*	*-2.207*
*Tff3*	*0.184*	*-1.790*
*Muc2*	*0.112*	*-2.141*

Differentially expressed genes (DEGs) altered with claudin-1 overexpression, with at least 1.5 fold change, involved in (A) Wnt Signaling (B) Notch-signaling or (C) Inflammation/Immune response (n = 3 mice in each group).

**Figure 4:**
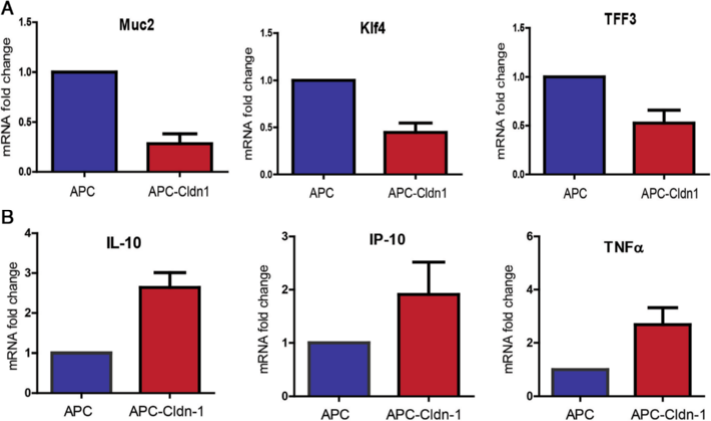
**APC-Cldn1 tumors have decreased mucosal defense and increased inflammation.** RNA was isolated from tumors of APC^Min^ and APC-Cldn1 mice and was utilized for qRT-PCR analysis of the **(A)** mucosal defense genes Muc2 (p = 0.0022), Klf4 (p = 0.0031), and Tff3 (p = 0.0653) and **(B)** inflammatory genes (n = 3 mice each group). Values are mean ± S.E.M.

Additionally, Notch target genes known to regulate mucosal defense were downregulated in APC-Cldn1 tumors. To validate mRNA expression of these genes, we performed qRT-PCR analysis (Figure 4A). Mucin-2, a primary component of the goblet cells and previously shown by us to be downregulated with claudin-1 overexpression [5], was also significantly down-regulated (p = 0.0022) in APC-Cldn1 tumors. Kruppel-like factor 4 (Klf4) and Trefoil factor 3 (Tff3), regulators of the goblet cell development and mucosal defense, were also decreased in APC-Cldn1 tumors (p = 0.0031, 0.0653). Studies assessing the effect of the loss of Muc2 or/and Klf4 in the regulation of APC mediated tumorigenesis have shown an increase in colon tumorigenesis as a result of increased mucosal inflammation [13,14]. Additionally, we observed several genes (Ccl6, Clc28, Pla2g5, Il-23, and Il-1b) corresponding to activated immune response and increased inflammation in our microarray analysis. These observations suggested inflammation as a possible mechanism for observed tumor progression in the colon of APC-Cld1 mice. We performed qRT-PCR analysis for common cytokines upregulated during inflammation and observed a significant increase in IL-10 mRNA expression (p = 0.0139), with an accompanied increase in IP-10 and TNFα (2- and 3-fold increase, respectively) (Figure 4B). These immune regulators have been shown to be upregulated in response to systemic inflammation, most notably observed in diseases such as colitis.

### The inflammation-driven colon tumor growth is upregulated in APC-Cldn-1 mice

Colon tumorigenesis can be induced in APC^Min^ mice by the administration of the colitis-inducing DSS [15]. This chemical activates inflammation, which drives the development of colonic tumors in these mice. We have shown previously that Cld-1Tg mice are susceptible to DSS-colitis [5]. Therefore, we reasoned that inflammation-driven colon tumor growth would also be upregulated in APC-Cldn-1 mice. To test, we treated APC^Min^ and APC-Cldn1 mice with 2% DSS drinking water for 5 days. Subsequently, mice were given normal drinking water and allowed to recover for 2 weeks until sacrifice. Endoscopic imaging was performed on each mouse on days 5, 8, and 12 following DSS administration, which showed increased inflammation and tumor development as early as day-5 post DSS-administration in APC-Cldn1 mice (Figure 2A) compared to APC^Min^ mice where colon tumor appeared around day-8. The total number of colonic tumors at the time of sacrifice showed that APC-Cldn1 mice developed more (p = 0.022) tumors than APC^Min^ mice (Figure 2B and C). Also, the tumors appeared larger in APC-Cld1 mice as compared to APC^Min^ mice. Combined together, we concluded that claudin-1 expression decreased tumor latency to increase tumor growth in the DSS-APC model of colon tumorigenesis.

### Constitutive Claudin-1 expression with APC mutation leads to adenoma associated-increased permeability and comprised barrier function

**Figure 5:**
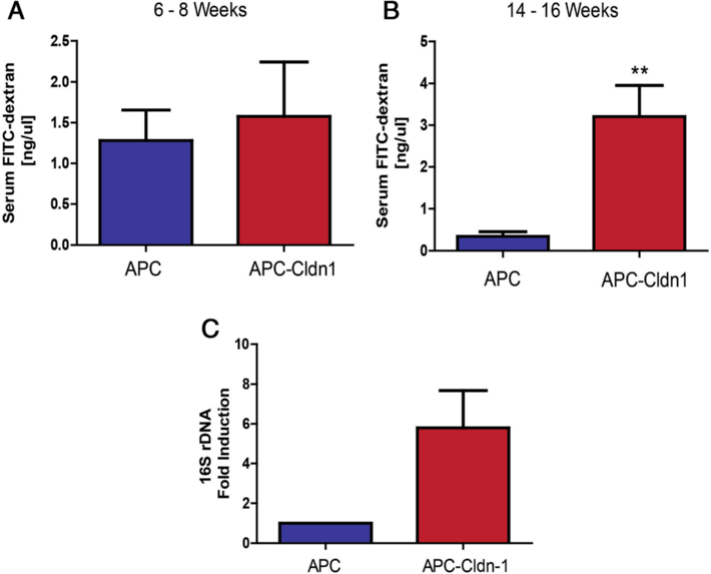
**APC-Cldn1 mice demonstrate increased colonic permeability and bacterial translocation into the mucosa.** Permeability was determined to FITC-dextran *via* rectal administration **(A)** at 6 - 8 weeks (n = 6) and **(B)** 14–16 weeks of age in APC and APC-Cldn1 mice (n = 7). **(C)** qRT-PCR analysis of 16S rDNA in the distal colon of APC and APC-Cldn1 mice at 16 weeks of age (n = 3 mice in each group). Values are mean ± S.E.M. *p < 0.05.

Recently, it was demonstrated that the barrier defect plays an important role in colon tumor progression by promoting inflammation [9]. Of note, APC mice between the ages of 12 to 14 weeks have increased permeability compared to age-matched wild type mice which is attributed to adenoma development [16]. Importantly, we have previously demonstrated that mucus barrier is compromised in Cld-1TG mice due to suppressed goblet cell differentiation [5]. Considering the advanced and increased colonic tumor burden in APC-Cld-1 mice (*versus* APC^Min^ mice), we also decided to assess mucosal permeability in these mice by measuring the permeability to FITC-labeled dextran. We observed an increased FITC-dextran permeability as early as approximately 8 weeks of age in APC-Cldn1 mice, with a significant increase (3-fold) observed at 14-16 weeks (Figure 5A,B), the age at which tumors were found to be present in the colon, compared to the APC^Min^ littermates. Increased gut permeability has been frequently linked to the increased microbial translocation [17,18]. Therefore, using genomic DNA isolated from 16 week old mice, we also measured potential microbial translocation into the mucosa of APC-Cldn1 mice via PCR analysis using primers for bacterial specific 16S rDNA, which demonstrated a significant increase (p = 0.0129) in APC-Cldn1 mice (Figure 5C).

**Figure 6:**
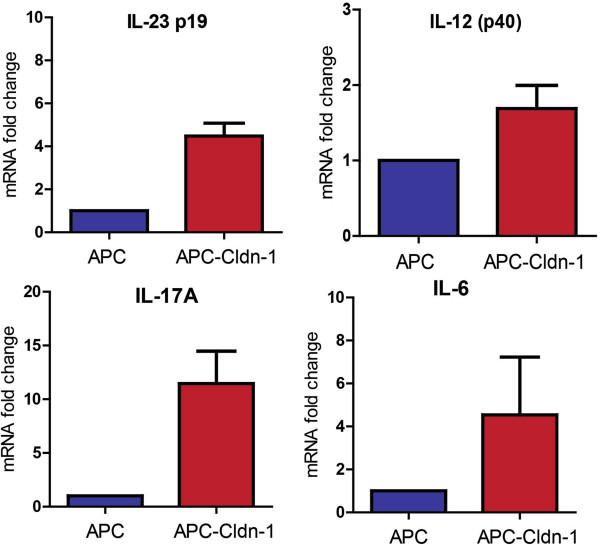
**IL-23 signaling is upregulated in colon tumors of APC-Cldn1 mice.** RNA was isolated from tumors of APC^Min^ and APC-Cldn1 mice and was utilized for qRT-PCR analysis of the IL23 pathway associated genes (IL-23, IL-12p40, IL-17A and IL-6). Values are mean ± S.E.M. (n = 3 mice in each group).

Recent studies have shown that increased inflammation, specifically IL-23/IL-17 signaling, contributes to the colon tumor development due to increased permeability and bacterial translocation [9]. Additionally, presence of IL-23 facilitates bacterial induced colitis [19]. Therefore, we assessed our model for potential upregulation of IL-23 signaling, and indeed, microarray analysis showed a significant increase in IL23a cytokine (Table 2). Validation using qRT-PCR also showed a significant increase (p = 0.0022) in IL23 specific p19 subunit (Figure 6). The downstream targets IL17A (p = 0.0442) and IL6 (4-fold induction) were also upregulated in APC-Cldn1 mice. Taken together, these results suggested that increased claudin-1 expression causes an increase in intestinal permeability in APC^Min^ mice. The increased gut permeability allows increased bacterial translocation, which in turn upregulates the IL23/IL-17 signaling to promote colon tumorigenesis.

## Discussion

Claudin-1 overexpression has been frequently observed in colon cancer, and mucosal inflammation, however the significance of its upregulation is not clearly understood. We utilized the APC^*Min*^ model of colon cancer to identify the role of claudin-1 in tumorigenesis and were able to determine that claudin-1 overexpression contributes to colon tumor growth and progression. The APC mice have a limited life span, which is often attributed to the tumor burden of the small intestine and cachexia that develops as a result. Considering the advanced nature of the intestinal tumors and increased colonic tumor burden in APC-Cldn1 mice, we believe that the advanced and increased tumors contributed to the decreased survival of APC-Cldn1 mice. However considering the key role of inflammation in tumor progression in our mice, its contribution to decreased survival can’t be ruled out.

The Wnt- and Notch-pathways have important roles in normal colonic development and are known to be dysregulated in intestinal diseases, most notably colorectal cancer. Claudin-1 is a known target of Tcf/Lef signaling however also seems to participate in the potentiation of this pathway as we have seen further upregulation of Wnt signaling in the APC-Cldn1 tumors. This also supports our previously published data of claudin-1 regulating E-cadherin expression through modulation of Wnt/β-catenin activity [20]. It is of interest to note that Wnt-target genes that are upregulated with claudin-1 overexpression are involved in stem cell maintenance (Figure 3C), as opposed to those that are well known oncogenes, cyclin D1 and c-myc, of which we observed no change in mRNA expression (data not shown). CD44 has been shown to be a marker of cancer stem cells and responsible for conferring tumorigenic properties to cells [21,22]. Lgr5 is an established intestinal stem cell marker and has also been shown to regulate tumorigenic capacity of colon cancer cells [23,24]. Although not identified as a stem cell marker, Axin2 is an established target of Wnt signaling and contributes to colon cancer cell invasion [25]. Further studies would establish whether claudin-1 could directly regulate expression of these genes.

In previous studies we showed that claudin-1 increases Notch-activity. We were able to detect increased Notch activity as measured by Hes1 and Math1 mRNA expression (Figure 3D). As crosstalk between the Notch- and Wnt-pathways is not a new concept, it is possible that claudin-1 could rest at the hub of this interaction. The microarray presented several shared genes (Table 2) between these pathways that were upregulated in tumors from APC-Cldn1 mice. Additionally, Lgr5 expression, which was increased by claudin-1 overexpression, can regulate Notch-activity [24].

We have shown in Cld-1-Tg mice and colon cancer cell lines that increased claudin-1 expression can increase Notch signaling with downregulation of expression of mucosal defense genes Muc2, KLF4 and Tff3 [5] and in this study microarray data and qRT-PCR analysis confirmed downregulation of these genes in the tumors of APC-Cldn1 mice. These genes, known to be important in the protection against inflammation and luminal antigens, have also been shown to be important in the protection against tumorigenesis. Klf4 expression can regulate tumor growth in mouse xenograft studies and tumor number in APC mediated tumorigenesis [14,26]. Similarly, Tff3 expression can also regulate tumor growth [27]. Muc2 deficient mice, known to develop spontaneous colitis, have been shown to robustly increase colon tumorigenesis when combined with APC mutation [13]. Interestingly, partial loss of Muc2 contributed to the tumor development in APC^Min^ mice in a fashion similar to APC-Cld-1 mice. These studies support a postulation that claudin-1 expression upregulate Notch signaling to regulate defense gene expression and their loss contributes to claudin-1 mediated tumorigenesis. Additionally these findings provide further support of a potential role for inflammation in claudin-1-mediated colon cancer progression. Of interest, we have recently demonstrated that constitutive claudin-1 expression induces Notch signaling which in turn suppresses goblet cell differentiation and muc-2 expression to compromise the mucus barrier and thus increase susceptibility to colitis in Cld-1Tg mice [5]. We predict that the compromised mucus barrier along with increased permeability due to the early onset of the adenomas help facilitate increased translocation of the luminal bacteria and microbial products into the mucosa of APC-Cld-1 mice, which in turn promotes inflammation, a driver of colon tumor progression. We indeed confirmed increased expression of cytokines commonly upregulated during inflammation in APC-Claudin-1 tumors (Figure 4B).

Sporadic cancer and colitis-associated cancers are frequently studied as two distinct processes separated by their initiating stimulus, loss of the APC or inflammatory bowel disease, respectively. Studies, including this work, have highlighted a cooperation of the two, beyond genetic manipulation of specific inflammatory mediators [9,13,28,29]. This suggests that whereas inflammation is generally thought to be a result of host response to tumor development in "hereditary" development of colon cancer, it may actually function to fuel tumor progression. We employed a model that accelerates the formation of colonic tumors in APC^Min^ mice to further examine the role of claudin-1 in tumorigenesis. With this model we were able to track tumor development in both APC^Min^ and APC-Cldn1 mice in a shorter amount of time. Colon tumors were significantly increased and we also observed that in this model APC-Cldn1 mice developed tumors earlier than APC^Min^ mice, further confirming our results from the sporadic model.

Loss of the mucosal defense genes and the resulting increased inflammation are factors that can be regulated by a breech in barrier dynamics. The role of claudin-1 in the regulation of barrier function was established shortly after its discovery. Here we show that claudin-1 overexpression in APC^Min^ mice induces mucosal permeability by inducing early adenoma formation. This change in permeability along with mucus barrier defect in these mice allows commensal bacteria to freely flow in to the intestinal mucosa. Indeed, it has been shown that bacteria can contribute to colon tumorigenesis [30]. Specifically, APC mice housed in a germ-free environment produced less tumors than those placed in normal housing conditions [31]. In accordance, APC-Cldn1 mice exhibited increased bacterial translocation and colon tumors, further supporting a role for the aid of commensal bacteria in the regulation of sporadic colon tumorigenesis.

Recently, studies involving a separate model of APC-Cre-mediated colon tumorigenesis showed that tumor formation was mediated by IL-23 signaling in response to barrier defect and increased bacterial products [9]. Indeed we observed increased IL-23 and its downstream targets, IL-17 and IL-6. Since IL-23 signaling is activated in response to bacteria, it is possible in this model that inflammation arises downstream of claudin-1 activation in response to increased bacterial translocation. It is also of interest to note that supernatants of colonic ex vivo explants from *Muc2/APC* mice had increased IL-23 secretion [13]. Additionally, many of the genes (Lcn2, Ccl28, and CCl6) found to be upregulated in the microarray of APC-Cldn1 mice were also found to be upregulated in the microarray of *Muc2/APC* mice [13].

Studies into the role of tight junction proteins in cancer have focused on their role in mechanical aspects of tumorigenesis, i.e., migration and invasion, which is not surprising considering the classical function of these proteins. It is ideal to think of alterations in tight junctions affecting latter stages of tumorigenesis as it relates to loss of polarity and contribution to EMT-like changes, thus facilitating metastasis. Our work shows a causative role of claudin-1 in earlier stages tumorigenesis. We have shown that increased expression of a tight junction protein can cause early adenoma formation which in turn causes enhanced permeability, increased bacterial translocation and thus inflammation with induction of IL-23 signaling to increase colonic tumorigenesis. This observation suggests claudin-1 has an active role in progressing tumorigenesis as opposed to being altered as a result. It is still unclear as to the sequence of events during tumorigenesis in relation to the order of inflammation and claudin-1 upregulation. It is possible to hypothesize a feedback loop may exist that maintains elevated inflammation. Claudin-1 expression has been shown to be regulated by cytokines [6,32], and here we have shown that claudin-1 can mediate inflammation through a mechanism involving reduced Muc2 and increased bacterial translocation. Further studies will investigate the specific mechanism by which claudin-1 upregulates IL-23 signaling beyond bacterial upregulation.

## Conclusion

This study identifies an essential role of claudin-1 protein in the regulation of colorectal cancer by upregulating Notch- and Wnt-signaling and mucosal inflammation.

## Methods

### Mice

To obtain APC^Min/+^-Villin-Cldn1Tg mice (APC-Cldn1), APC^Min^ males purchased from Jackson Laboratories (Bar Harbor, Maine) were bred with Villin-Claudin-1-Tg females. Claudin-1 overexpression was assessed by PCR as described previously [5] on genomic DNA isolated from tail snips using DNA isolation buffer (Viagen Biotech; Los Angeles, CA). Identification of the mutated APC allele was performed using a modified protocol from Jackson Laboratories. APC and APC-Cldn1 littermates were monitored for signs of morbidity including hunched posture, anemia, and body weight loss and sacrificed according to the guidelines of Vanderbilt University Institutional Animal Care and Use Committee (IACUC). Accelerated tumorigenesis was induced by administering 10-week-old mice dextran sulphate sodium (DSS 2%) in their drinking water. Mice were monitored throughout duration of the experiment as described previously [5], and examined intermittently via colon endoscopy.

### Tissue processing

As described previously, the colon and the small intestine were dissected and flushed with PBS, opened flat and formalin fixed using the Swiss roll method. Further processing was performed by the Vanderbilt Translational Pathology Shared Resource Core. Distal and proximal sections of the colon were snap-frozen and stored at -80°C for further analysis. Where applicable, colonic tumors were quantified. Tumors were either isolated and frozen for further analysis, or left in the colon to be processed for embedding and H&E staining.

### Tumor size measurements

Tumor area was calculated using Axiovision 4 digital imaging processing software (Release 4.8.1, Carl Zeiss Imaging) by outlining tumors of three to four mice each of APC and APC-Cldn1 mice.

### Immunohistochemistry

Immunostaining of the paraffin-embedded tissues was performed as described previously [5] using VectaStain ABC kit (Vector Laboratories) and the indicated antibodies. Images were obtained using a Zeiss light microscope.

### RNA isolation and microarray analysis

Total RNA was isolated from tumors excised from the colon of APC and APC-Claudin-1 mice using Qiagen RNAeasy Mini kit with DNAse digestion step performed. The integrity of the RNA was determined by performing formaldehyde gel electrophoresis. Samples displaying two bands, corresponding to the 18S and 28S subunits, and having an A260/A280 of ~1.8 were used for experiments. Total RNA was isolated from snap-frozen tumors, as described above, and RNA integrity was measured. Samples were submitted to the Vanderbilt Microarray Shared Resource.

### Quantitative reverse transcription-PCR

Total RNA (1ug) of each sample was reverse transcribed using the iScript cDNA Synthesis Kit (Bio-Rad). Each qRT-PCR reaction contained SYBR Green Master Mix, the indicated primer sets and 25 ng of cDNA. Samples were loaded in triplicates on 96 well plates and run on a Bio-Rad iCycler. Ct values were utilized to calculate fold change and normalization was performed using beta-actin.

### Permeability assay

Assessment of intestinal permeability to 4 kDa FITC-dextran was performed as described previously by rectal administration [5].

### Bacterial translocation

Translocation of bacteria was assessed by detecting the amount of bacterial 16S rDNA using qRT-PCR and specific primers for conserved regions of the bacterial 16S rDNA [33]. Genomic DNA was isolated from the distal colon of three mice per group and 25 ng was used per reaction. The mouse *Selp* gene was utilized as normalization gene.

### Statistics

Statistical analyses were performed using Graphpad Prism software (San Diego, CA) for t-test analysis where comparisons between two groups were involved. SPSS software (College Station, TX) was utilized for analyses of Logistic regression (for binary outcomes) and Chi^2^ (for categorical). *P* values less than 0.05 were considered significant.

## Abbreviations

APC: Adenomatous polyposis coli gene; IEC: Intestinal epithelial cell; FAP: Familial adenomatous polyposis.

## Competing interests

The authors declare that they have no competing interest.

## Authors’ contributions

JLP: Conception and design, acquisition of data, analysis and interpretation of data, writing of the manuscript, RA: Acquisition of data, analysis and interpretation of data. AAB: Acquisition of data, analysis and interpretation of data. MKW: analysis and interpretation of data, ABS: Obtained funding, conception and design, manuscript writing and PD: Obtained funding, conception and design, study supervision, writing of the manuscript. All authors read and approved the final manuscript.

## Supplementary Material

Additional file 1: Figure S1 Expression of Claudin-1 in APC-Cld1 mice compared to APC mice. (A) Immunoblot of normal colon tissue from APC and APC-Cldn1 mice (n = 3) shows robust expression of claudin-1 in APC-Cldn1 mice. (B). Representative immunostaining images of claudin-1 expression in APC and APC-Cldn1 colon shows increased expression localized to the membrane of APC-Cldn1 mice. Click here for file

Additional file 2: Figure S2 Quantification of Small Intestine Adenomas. Adenomas of small intestine from APC and APC-Cldn1 mice were quantified (N = 10). Click here for file

Additional file 3: Table S1 Comparative Histological Analysis of the Small Intestine Adenomas. Click here for file

Additional file 4: Figure S3 Apoptosis is not altered between the colon tumors from APC^Min^ and APC-Cldn1 mice. Immunostaining using anti-cleaved caspase-3 antibody was done using the colon adenomas from APC^Min^ and APC-Cldn1 mice. Click here for file
