# Supplementary figures and images for: Claudin-1 overexpression in intestinal epithelial cells enhances susceptibility to adenamatous polyposis coli-mediated colon tumorigenesis
Source: Mol Cancer. 2014 Jul 6;13:167. doi: 10.1186/1476-4598-13-167 (PMC4105545; doi:10.1186/1476-4598-13-167)

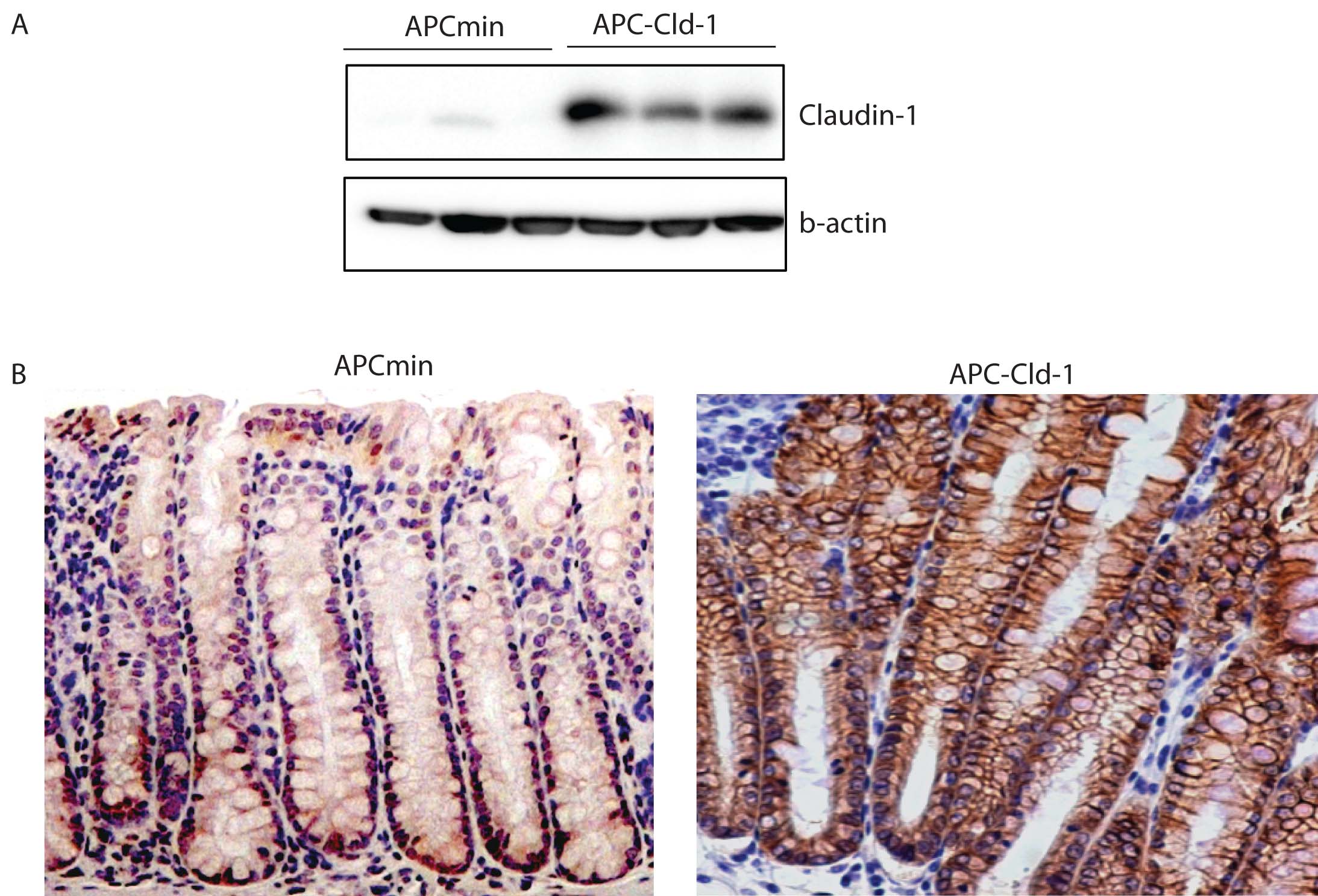

Supplement: Additional file 1: Figure S1 — Expression of Claudin-1 in APC-Cld1 mice compared to APC mice. (A) Immunoblot of normal colon tissue from APC and APC-Cldn1 mice (n = 3) shows robust expression of claudin-1 in APC-Cldn1 mice. (B). Representative immunostaining images of claudin-1 expression in APC and APC-Cldn1 colon shows increased expression localized to the membrane of APC-Cldn1 mice. [file 1476-4598-13-167-S1.jpeg]

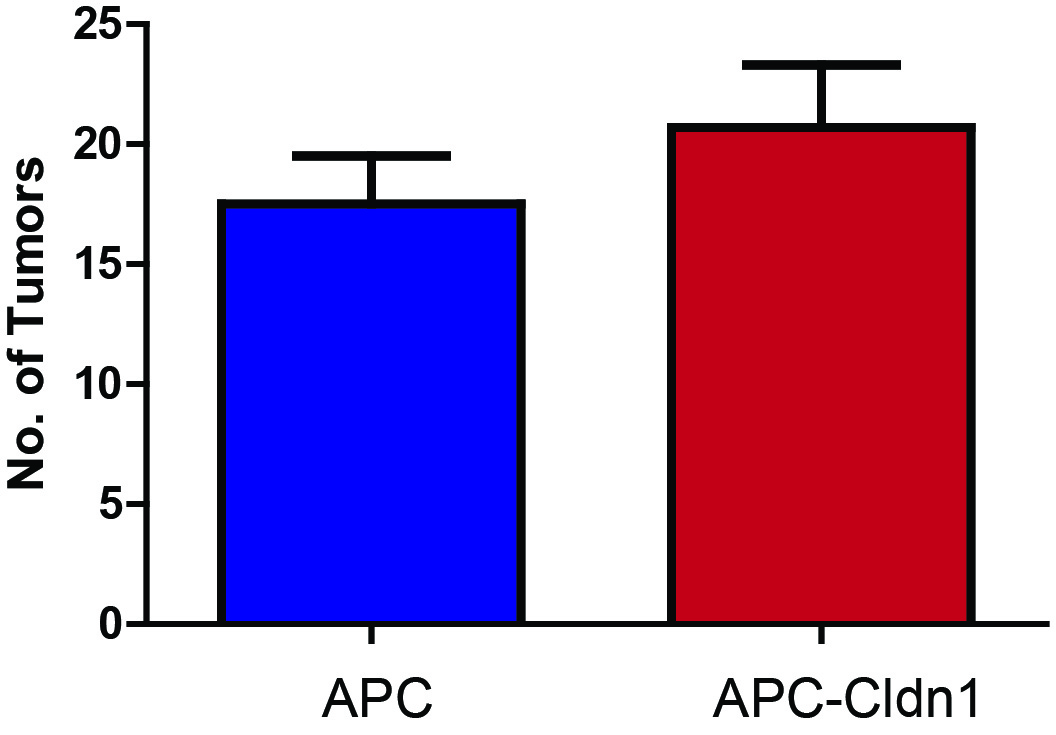

Supplement: Additional file 2: Figure S2 — Quantification of Small Intestine Adenomas. Adenomas of small intestine from APC and APC-Cldn1 mice were quantified (N = 10). [file 1476-4598-13-167-S2.jpeg]

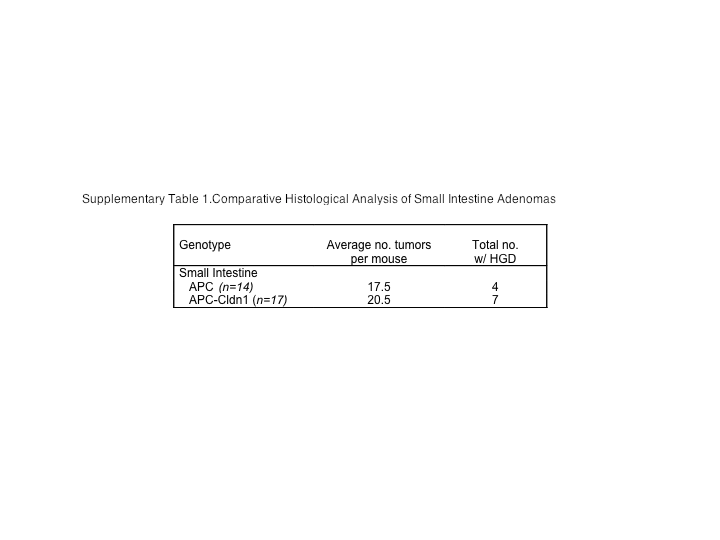

Supplement: Additional file 3: Table S1 — Comparative Histological Analysis of the Small Intestine Adenomas. [file 1476-4598-13-167-S3.tiff]

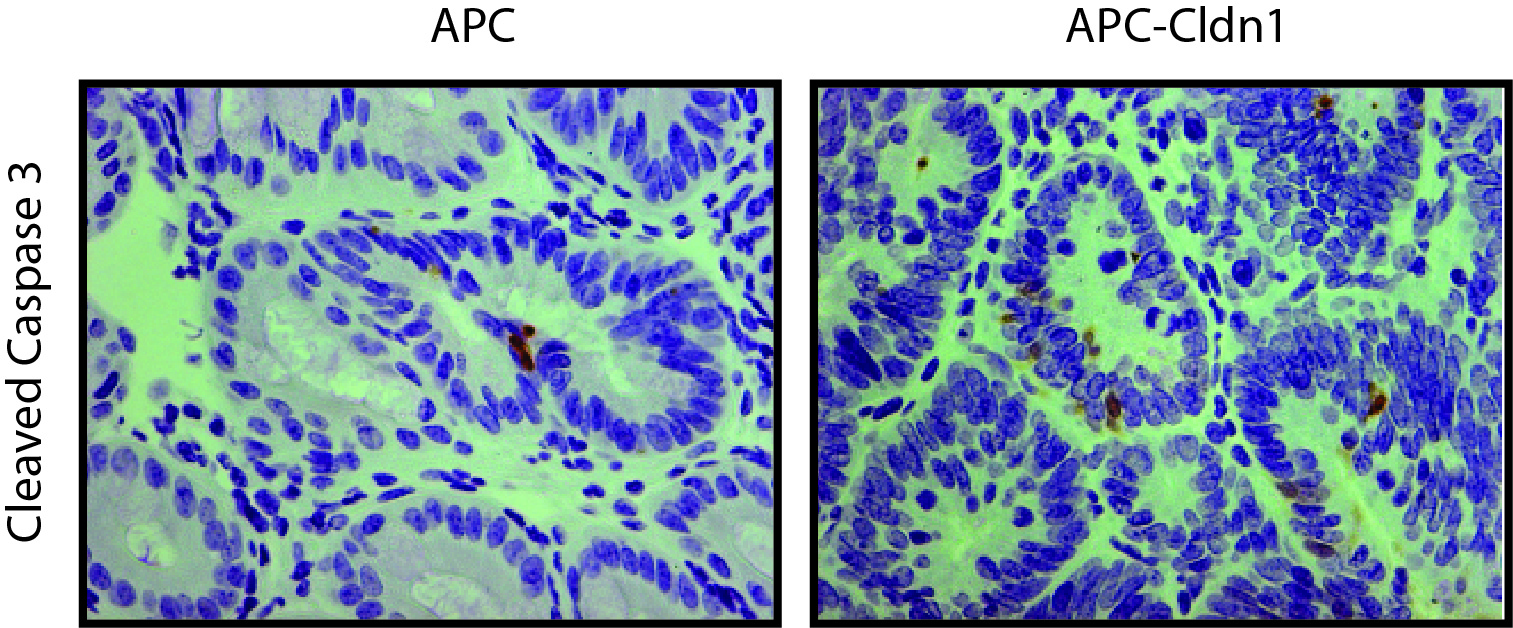

Supplement: Additional file 4: Figure S3 — Apoptosis is not altered between the colon tumors from APCMin and APC-Cldn1 mice. Immunostaining using anti-cleaved caspase-3 antibody was done using the colon adenomas from APCMin and APC-Cldn1 mice. [file 1476-4598-13-167-S4.jpeg]
